# The A673T mutation in the amyloid precursor protein reduces the production of β-amyloid protein from its β-carboxyl terminal fragment in cells

**DOI:** 10.1186/s40478-015-0247-6

**Published:** 2015-11-04

**Authors:** Asuka Kokawa, Seiko Ishihara, Hitomi Fujiwara, Mika Nobuhara, Minori Iwata, Yasuo Ihara, Satoru Funamoto

**Affiliations:** Department of Neuropathology, Graduate School of Life and Medical Sciences, Doshisha University, Kyotanabe, Kyoto, 610-0394 Japan; Department of Neuropathology, Graduate School of Brain Sciences, Doshisha University, Kizugawa, Kyoto 619-0225 Japan

**Keywords:** Alzheimer’s disease, Aβ, APP, γ-secretase, C99, Lipid raft

## Abstract

**Introduction:**

The A673T mutation in the amyloid precursor protein (APP) protects against Alzheimer’s disease by reducing β-amyloid protein (Aβ) production. This mutation reduced the release of the soluble APP fragment (sAPPβ), which is processed by β-secretase, suggesting a concomitant decrease in the β-carboxyl fragment of APP (C99), which is a direct substrate of γ-secretase for Aβ production. However, it remains controversial whether the level of C99 is significantly reduced in cells expressing APP that carry A673T as the cause of reduced Aβ production. Here, we investigated the effect of the A673T mutation in C99 on γ-cleavage in cells.

**Results:**

We found that the level of C99 in cells expressing APP A673T was indistinctive of that observed in cells expressing wild-type APP, although the release of sAPPβ was significantly reduced in the APP A673T cells. In addition, our reconstituted β-secretase assay demonstrated no significant difference in β-cleavage on an APP fragment carrying the A673T mutation compared with the wild-type fragment. Importantly, cells expressing C99 containing the A673T mutation (C99 A2T; in accordance with the Aβ numbering) produced roughly half the level of Aβ compared with the wild-type C99, suggesting that the C99 A2T is an insufficient substrate of γ-secretase in cells. A cell-free γ-secretase assay revealed that Aβ production from the microsomal fraction of cells expressing C99 A2T was diminished. A sucrose gradient centrifugation analysis indicated that the levels of the C99 A2T that was codistributed with γ-secretase components in the raft fractions were reduced significantly.

**Conclusions:**

Our data indicate that the A673T mutation in APP alters the release of sAPPβ, but not the C99 level, and that the C99 A2T is an inefficient substrate for γ-secretase in cell-based assay.

**Electronic supplementary material:**

The online version of this article (doi:10.1186/s40478-015-0247-6) contains supplementary material, which is available to authorized users.

## Introduction

Senile plaques are one of neuropathological hallmarks of Alzheimer’s disease (AD) and composed of β-amyloid protein (Aβ). Aβ is a small protein consisting of 38–43 residues that is produced from the amyloid precursor protein (APP) via sequential cleavage by β- and γ-secretases [1–5]. Longer forms of Aβ, such as Aβ42 and Aβ43, are prone to aggregation and are initially deposited in the brain [6]. Familial AD (FAD) mutations in APP, presenilin 1, and presenilin 2 increase the ratio of Aβ42/Aβ40. We found previously that FAD mutations preferentially produced a longer form of the APP intracellular domain (AICD49–99), rather than AICD50–99, and that the expression of Aβ48, as a counterpart of AICD49–99, resulted in an increase in Aβ42/Aβ40 ratio, as found for FAD mutations [7, 8].

Genetic analyses in AD provide not only insights into the molecular mechanisms underlying the pathogenesis of AD, but also a perspective regarding the prevention and cure of the disease. Recently, the A673T mutation in APP was reported as a novel protective mutation against AD that acts by reducing Aβ production [9]. Although this mutation seems to be restricted to Iceland and Nordic countries, this finding, together with FAD mutations, strongly supports the amyloid hypothesis [10–17]. The A673T mutation causes a substantial decrease in the level of the soluble APP fragment processed by β-secretase (sAPPβ) [9, 18]. It is reported that the level of the β-carboxyl fragment of APP (C99, also known as βCTF and CTFβ), which is a direct substrate of γ-secretase for Aβ production, in cells expressing APP A673T was one fourth of that detected in cells expressing APP wild-type (WT), which suggests the concomitant decrease of C99 as a counterpart of sAPPβ [18]. Conversely, Benilova and colleagues reported that cells expressing APP A673T exhibited no significant difference in the levels of C99 compared with cells expressing WT APP, in spite of a nearly two hundredth reduction in sAPPβ levels [19]. Thus, it is unclear whether a decrease in sAPPβ leads to a concomitant decrease in C99 as a cause of reduced Aβ production in cells expressing APP A673T.

Recently, we found that γ-secretase preferentially cleaved substrates with a short ectodomain, which supports the idea that γ-secretase recognizes the amino terminus of the substrate [20, 21]. The A673T mutation (i.e., A2T; in accordance with the Aβ numbering) lies within the amino terminus of the C99 substrate. It remains unknown whether the A2T substitution in C99 affects γ-secretase-dependent cleavage in cells. In this study, we examined the direct effect of A2T substitution in C99 on γ-secretase-dependent cleavage.

## Materials and methods

### Antibodies

Cell lysates and conditioned media were subjected to western blotting using the following antibodies: 6E10 (total Aβ; Covance), 82E1 (total Aβ; IBL), E50 (total Aβ), BA27 (Aβ40), BC05 (Aβ42), and anti-GFP (Santa Cruz) [22, 23].

### Constructs

The C99-FLAG tag-coding region was fused to the APP signal peptide. Additional Asp and Ala residues were inserted between the APP signal peptide and C99-FLAG, which allowed precise cleavage at the β-cleavage site, generating C99 and Aβ species that start from Asp-1 [8, 24]. This C99-FLAG coding region or full-length APP region was inserted prior to the internal ribosome entry site (IRES) of pMXIG. This construct allows cells to express GFP as an internal standard. For pulse-chase analysis, the full-length APP-FLAG tag-coding region was inserted into pcDNA4/TO (Invitrogen) and transfected in T-Rex CHO cells (Invitrogen).

### Pulse-chase analysis of APP processing

T-Rex CHO cells were cultured in 12-well plates and transfected with pcDNA4/TO carrying full-length APP (WT or A673T) [25]. Twenty-four hours later, cells were treated with tetracycline at a concentration of 1 μg/mL for 4 h, to express APP. Cells were washed twice in tetracycline-free medium and incubated in the medium for 24 h. Cells and media were collected every 4 h and subjected to western blotting, to visualize and quantify the levels of APP, sAPPβ, sAPPα, C99, C83, and Aβ.

### Secretase assays

A γ-secretase assay and coimmunoprecipitation analyses were performed as described previously [21, 26]. Briefly, the C99-FLAG substrate was expressed in sf9 cells and purified using anti-FLAG M2 agarose beads. The C99-FLAG substrate was incubated with a γ-secretase fraction for 4 h and subjected to western blotting. For coimmunoprecipitation of C99 with γ-secretase components, the C99-FLAG substrate was immobilized on anti-FLAG M2 magnetic beads and incubated with a γ-secretase fraction. For β-secretase assay, the human APP fragment 633–685 (numbering from APP751) fused with N-terminal Myc and C-terminal FLAG tags (referred to as APP633–685-FLAG) was expressed in *Escherichia coli* BL21 cells and affinity purified using ANTI-FLAG M2 beads [21]. The purified APP633–685-FLAG (500 nM) was incubated with β-secretase (Sigma) for 4 h, according to the manufacturer’s instructions. β-Cleaved C-terminal fragments (Aβ33-FLAG) from APP633–685-FLAG were visualized and quantified using the E50 antibody.

### Cell-free γ-secretase assay

Cells were cultured in Dulbecco’s modified Eagle’s medium (Sigma) supplemented with 10 % FBS (Invitrogen) and penicillin/streptomycin (Invitrogen). Harvested cells were homogenized in Buffer A (20 mM PIPES, pH 7.0, 140 mM KCl, 0.25 M sucrose, and 5 mM EGTA) with a glass/Teflon homogenizer. Postnuclear supernatants were subjected to ultracentrifugation at 100,000 g for 1 h. The pellets were resuspended in Buffer A at a protein density of 2.5 mg/mL and defined as microsomal fractions [27]. Microsomal fractions from C99 WT and C99 A2T cells were incubated at 37 °C and lipids were extracted with chloroform/methanol before western blotting.

### Isolation of CHAPSO-insoluble fractions

The CHO homogenate was adjusted to 40 % sucrose and centrifuged on a discontinuous sucrose gradient for 20 h at 4 °C using an SW 41 Ti rotor (Beckman) [28, 29]. After centrifugation, the homogenate was fractionated into 12 fractions and subjected to western blotting using the following antibodies: N1660 (Nicastrin; 1/3000 in TBS containing 0.1 % Tween; Sigma), anti-Aph-1a C-term antibody (Aph-1; 1/1000 in TOYOBO Can Get Signal; Covance), anti-PS1–CTF antiserum (Presenilin 1 CTF; 1/3000 in TBS containing 0.1 % Tween; gifts from Drs. T. Iwatsubo and T. Tomita, The University of Tokyo), anti-Pen-2 antibody (Pen-2; 1/3000 in TBS containing 0.1 % Tween; a gift from Dr. A. Takashima, National Center for Geriatrics and Gerontology), anti-caveolin antibody (caveolin-1; 1/1000 in TBS containing 0.1 % Tween; Santa Cruz), and anti-flotillin antibody (flotillin-1; 1/1000 in TBS containing 0.1 % Tween; BD).

## Results

### Effect of the A673T mutation on C99 levels in cells

**Fig. 1:**
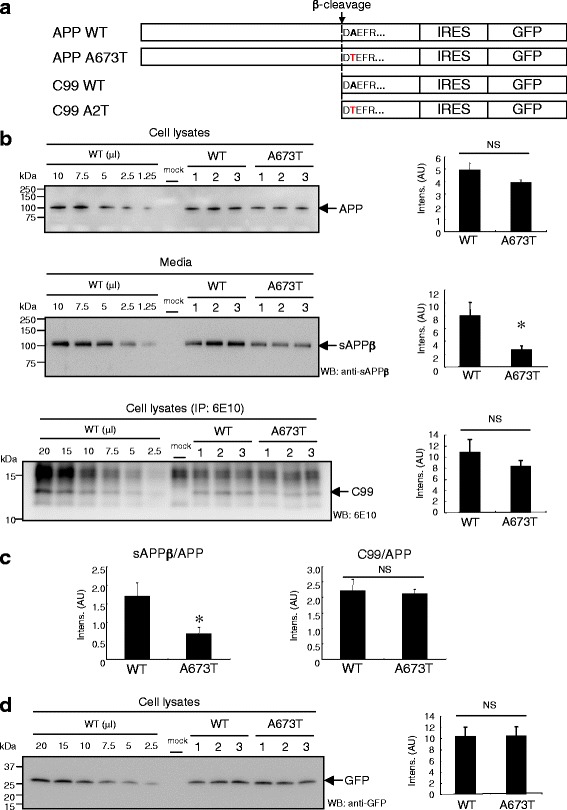
The A673T substitution in APP decreased sAPPβ secretion, but not C99. Constructs used in this study. The APP695- or C99-coding fragment was inserted prior to IRES-GFP in pMXIG (**a**). APP A673T cells released one third of the levels of sAPPβ compared with those of wild-type APP. However, C99 levels in APP A673T cells were statistically indistinguishable from those of APP WT cells (**b**). Levels of sAPPβ and C99 were normalized for amount of APP (**c**). C99 level in APP A673T cells was almost identical to that in wild-type APP cells, as well as from internal standard GFP expression levels (**d**). Data represent means ± SD of three independent experiments. NS, not significant; * *P* < 0.05 (unpaired *t*-test)

**Fig. 2:**
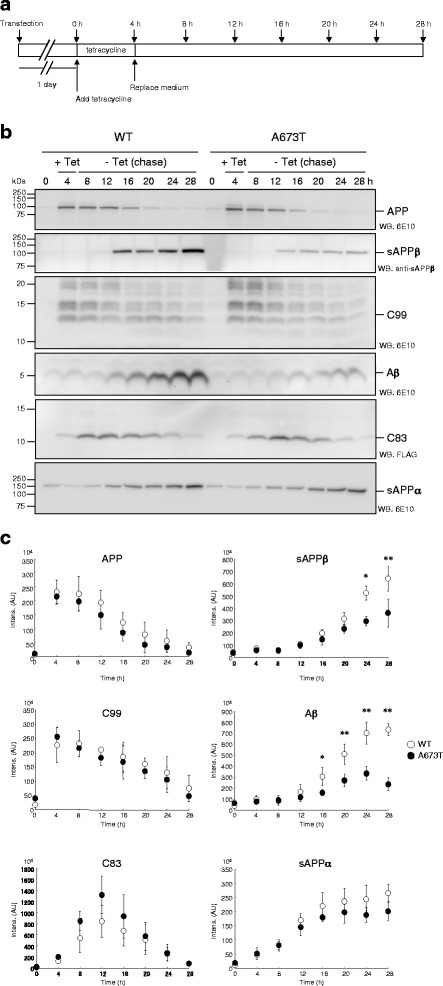
Pulse chase analysis of APP processing. Schematic diagram of pulse chase analysis of APP processing (**a**). CHO cells were transfected with APP WT or APP A673T construct and treated with 1 μg/ml tetracycline for 4 h. After replacing media, cells and media were collected at the time indicated. Detection of APP, sAPPβ, C99, Aβ, C83 and sAPPα by Western Blotting (**b**). C99 was barely detected by using anti-FLAG antibody, although robust C83 bands were visualized. Quantitative analyses of APP, sAPPβ, C99, Aβ, C83 and sAPPα (**c**). Levels of sAPPβ and Aβ were reduced in APP A673T cells compared with those in APP WT cells, while levels of APP and C99 were almost similar to those of APP WT cells. It is interesting to note that generation rate of C83 is distinct from those of APP and C99. Data represent means ± SD of three independent experiments. Open circle, WT; closed circle, A673T. *P < 0.05; **P < 0.01 (unpaired *t*-test)

The A673T mutation in APP reduced the release of sAPPβ [9]. We also observed that the secretion of sAPPβ into the medium was diminished in CHO cells expressing APP A673T (APP A673T cells) (Fig. 1); however, the levels of C99 carrying A2T (C99 A2T) in APP A673T cells were not affected, which was in agreement with the study reported by Benilova and colleagues (Fig. 1b and c) [19]. To assess the rates of sAPPβ, C99, and Aβ generation from APP A673T, we performed a pulse-chase analysis of APP processing using the Tet-ON expression system (Fig. 2a). Although the generation rates of sAPPβ and Aβ in APP A673T cells were attenuated, the generation rate of C99 in these cells was comparable to that observed in APP WT cells (Fig. 2b and c). Our observation was discrepant with a previous report [18]. To explain this disagreement, we focused on the immunoreactivity of 82E1, the antibody used in the ELISA system in the previous study. 82E1 was developed for amino-terminus-specific Aβ detection [22]. Our western blot analyses indicated that 82E1 failed to detect C99 A2T and Aβ A2T (Additional file 1: Figure S1). To confirm further the immunoreactivity of 82E1 on C99 A2T in A673T cells, we performed immunoprecipitation of C99 A2T using 6E10 and 82E1. 6E10 captured C99, regardless of the substitution, whereas 82E1 failed to bind to C99 A2T (Additional file 1: Figure S2A). We also immunoprecipitated C99 A2T in the APP A673T cells with anti-FLAG M2 antibody recognizing carboxyl terminal of APP-FLAG and detected C99 A2T in APP A673T cells as well as C99 WT in APP WT cells with anti-FLAG M2, but not 82E1 (Additional file 1: Figure S2B). Our data demonstrated that the level of C99 in APP A673T cells was comparable to that detected in APP WT cells, despite a significant reduction in sAPPβ, suggesting that the level of C99 in APP A673T cells is insufficient to explain the reduced level of Aβ secreted by these cells.

### Effect of the A673T mutation on the β-cleavage of the APP fragment

**Fig. 3:**
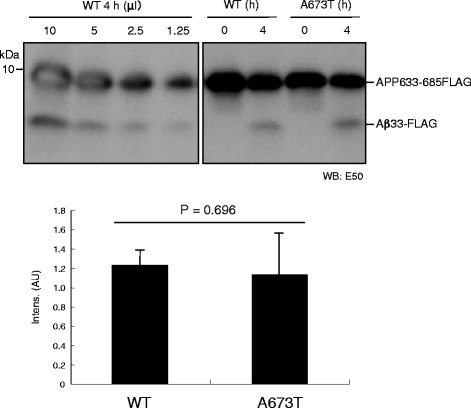
β-Secretase-dependent cleavage of the APP A673T fragment. An APP751 fragment (633–685) fused with FLAG tag (APP633–686-FLAG) was incubated with β-secretase. The E50 antibody was used to visualize the equal amounts of Aβ33-FLAG that were produced by β-secretase from the WT of the APP633–686-FLAG substrate and its A673T mutant substrate. The reaction solution of the WT substrate was loaded onto a gel, as indicated by standard curve. Data are expressed as means ± SD of three independent experiments. An unpaired *t*-test detected no significant differences between substrates

The reduction in sAPPβ level observed in APP A673T cells suggests a reduced β-cleavage in APP A673T. However, we detected no significant reduction in C99 levels in APP A673T cells. This raises the possibility that the A673T mutation has no significant impact on the β-cleavage of APP. Although pioneer studies have demonstrated reduced β-cleavage of APP A673T, we revisited this issue [9, 18]. We previously established a reconstituted β-secretase assay using the APP 633–685 substrate [21]. In this study, we examined the effect of the A673T mutation on the β-cleavage of the substrate carrying the mutation in our assay system. As shown in Fig. 3, APP 633–685-FLAG carrying A673T exhibited a level of Aβ33-FLAG production that was equivalent to that of the WT in our assay. This indicates that the A673T mutation does not affect the β-cleavage of APP.

### Effect of the A2T substitution on C99 regarding Aβ production in cells

**Fig. 4:**
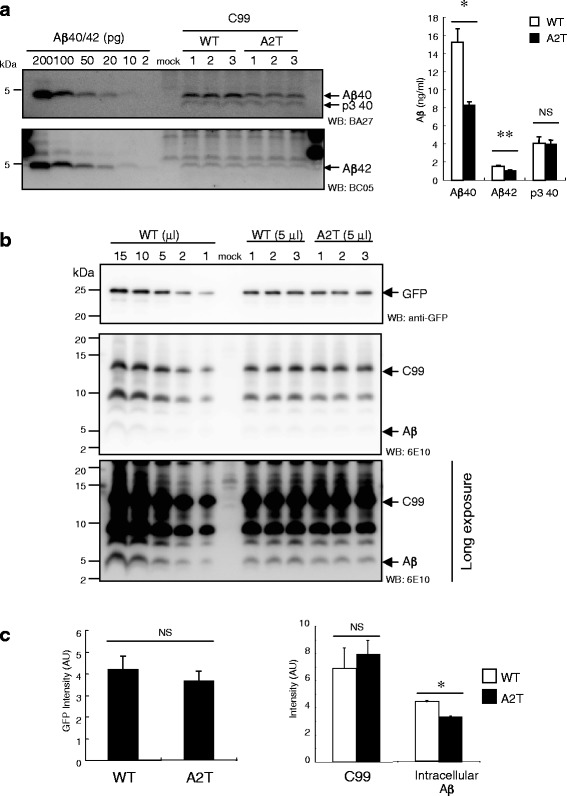
Conditioned media of C99 WT and C99 A2T cells were subjected to Western blotting to visualize and quantify the Aβ and p3 species. C99 A2T cells exhibited reduced levels of Aβ40 and Aβ42, but not of p3 40 (**a**). Lysates of transfectants were subjected to Western blotting, to visualize and quantify GFP, C99, and intracellular Aβ (**b**). The lysate of C99 WT cells was loaded into a gel, as indicated by standard curve. The levels of GFP and C99 in cells expressing C99 WT and C99 A2T were indistinctive, whereas intracellular Aβ levels were significantly reduced in C99 A2T cells. Data represent means ± SD of three independent experiments (**c**). NS, not significant. * *P* < 0.05 (unpaired *t*-test)

To uncover the molecular mechanism underlying the involvement of the A673T mutation in reduced Aβ production, we tested whether the A2T substitution in C99 directly suppressed Aβ production in cells (Fig. 1a). Importantly, CHO cells expressing C99 A2T (C99 A2T cells) released approximately half the levels of Aβ40 and Aβ42 into the medium in the case of the WT C99 (C99 WT cells), whereas p3 40 levels in the C99 A2T medium were not affected at all (Fig. 4a). The alteration of Aβ secretion by the expression of C99 A2T was observed even in other cell lines (Additional file 1: Figure S3). In addition, we found a significant reduction in intracellular Aβ in C99 A2T cells (Fig. 4b and c). These data demonstrate that the A2T substitution in C99 suppresses Aβ production in cells.

### No effect of the A2T substitution in C99 on its γ-cleavage in a membrane-soluble state

**Fig. 5:**
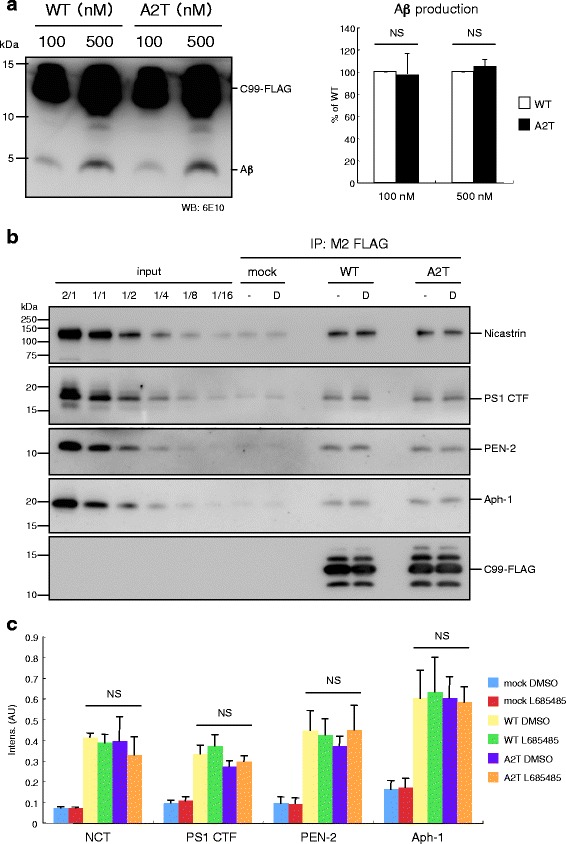
CHAPSO-solubilized γ-secretase assay. Recombinant C99-FLAG substrates were incubated with solubilized γ-secretase and subjected to Western Blotting to visualize and quantify levels of Aβ production (**a**). Aβ production from C99 A2T was indistinctive to that from C99 WT. Data represent means ± SD of three independent experiments. NS, not significant (unpaired *t*-test). Substrates were immobilized on anti-FLAG magnetic beads and incubated with CHAPSO-solubilized γ-secretase (**b**). After sufficient wash, the beads were subjected to Western Blotting. γ-Secretase interacted with C99 A2T as well as C99 WT. -, DMSO vehicle control; D, 1 μM DAPT. Quantitative analyses of interacted γ-secretase components (**c**). Data represent means ± SD of three independent experiments in the absence of 1 μM DAPT. NS, not significant (ANOVA, Scheffe’s post hoc test compared with WT DMSO)

The A2T substitution lies within the amino terminus of C99, which is recognized by γ-secretase [20, 21]. One may expect that γ-secretase would fail to recognize C99 A2T because of suppressed Aβ production. To test this possibility, we performed a CHAPSO-solubilized γ-secretase assay using the C99 A2T substrate, which allows free collision between the enzyme and substrate in the solution. Despite intensive analyses, we detected no significant reduction in Aβ production from recombinant C99 A2T in the solution (Fig. 5a). Moreover, a coimmunoprecipitation analysis revealed that the interaction between C99 A2T and γ-secretase components was not affected in the presence or absence of DAPT (Fig. 5b and c) [21].

### Effect of the A2T substitution on Aβ production in microsomal fractions

**Fig. 6:**
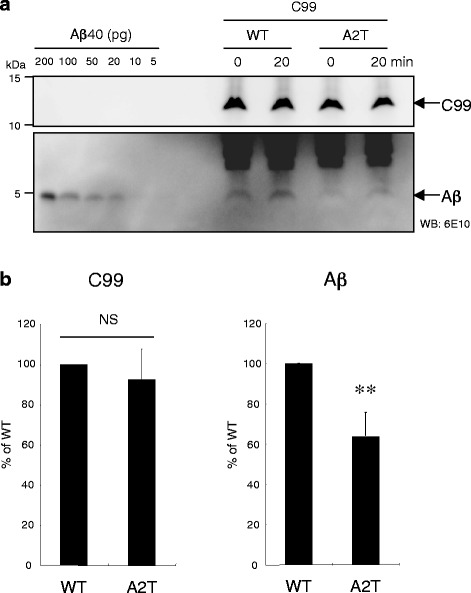
The microsomal fraction of C99 A2T was incubated and subjected to Western Blotting analysis (a). Aβ production from the microsomal fraction of C99 A2T cells was significantly reduced compared with that detected in C99 WT cells. Aβ production was determined by subtracting the amount of Aβ at 0 min from that at 20 min. Data represent means ± SD of four independent experiments (b). NS, not significant. **P < 0.005 (unpaired t-test)

Data regarding Aβ production in the CHAPSO-solubilized γ-secretase assay contradicted the results of the cell-based assay in terms of C99 cleavage by γ-secretase. One possible explanation for this discrepancy is the altered distribution of C99 A2T at the membrane. C99 A2T may be recognized by γ-secretase inefficiently in certain membrane compartments, resulting in the inhibitory effect of the A2T substitution on Aβ production in cells. To test this possibility, we performed a cell-free assay using the microsomal fraction of C99 A2T cells. We observed that C99 A2T yielded a significant decrease in Aβ production in the cell-free assay, although the levels of C99 A2T and C99 WT were almost the same (Fig. 6). These findings imply that the A2T substitution alters the subcellular distribution of C99, which leads to interference with the interaction between γ-secretase and C99 in certain membrane compartments.

### Altered distribution of C99 A2T in lipid rafts

**Fig. 7:**
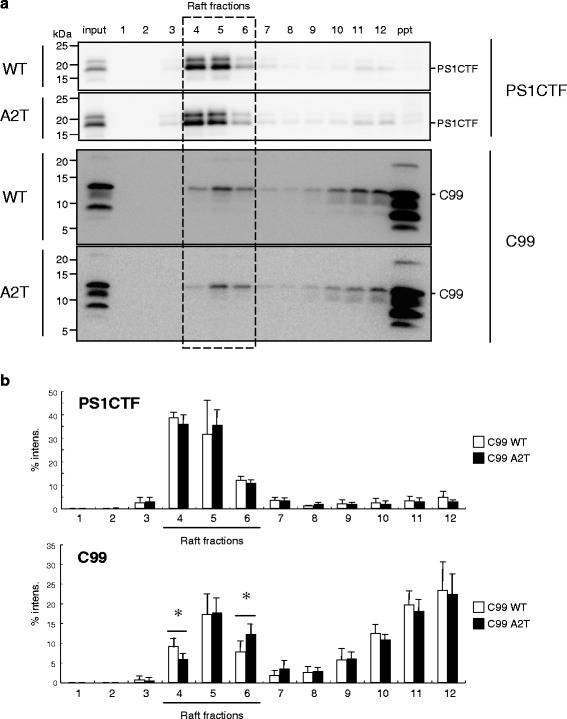
C99 A2T cells were treated with CHAPSO and fractionated using sucrose gradient centrifugation (**a**). The presenilin 1 carboxyl terminal fragment (PS1CTF) was enriched in raft fractions (#4 and #5). C99 was also distributed in these fractions, in part. However, C99 A2T distribution was significantly shifted into denser fractions (#5 and #6) compared with C99 WT (#4, #5, and #6) (**b**). Data represent means ± SD of three independent experiments. NS, not significant. *P < 0.05 (unpaired *t*-test)

Active γ-secretase accumulates in detergent-insoluble fractions (lipid raft) of membranes prepared by sucrose gradient centrifugation [30–32]. C99 also distributes to these fractions, in part. To investigate whether C99 A2T exhibits an altered distribution in lipid rafts, C99 A2T cells were treated with 1 % CHAPSO and subjected to sucrose gradient centrifugation as described previously [28, 29]. Importantly, the levels of the C99 A2T that was codistributed with γ-secretase components in the raft fractions were reduced significantly, although we observed no prominent difference between C99 WT and C99 A2T in membrane, cytosolic and nuclear fractions (Fig. 7; Additional file 1: Figure S4-S5). Our data suggest that the altered subcellular distribution of C99 can result in a moderate interaction between C99 and γ-secretase in cells, and in a reduction in Aβ production.

## Discussion

The A673T mutation in APP has been recognized as a protective variant of late-onset of AD and has been related to longevity in an Icelandic population [9]. Although this variant is extremely rare in non-Nordic countries, it is important to explore the mechanism of reduced Aβ production [14]. Biochemical and cell-based assays demonstrated reductions in the β-cleavage on an APP A673T fragment and in sAPPβ release from APP A673T cells, which suggests that reduced C99 production results in a reduction of Aβ production [9, 18]. In fact, β-cleavage has been a promising drug target for anti-AD therapeutics, to reduce C99 and Aβ [2, 33]. However, Benilova and colleagues observed no significant difference in the levels of C99 between APP WT and A673T cells, despite a substantial reduction in sAPPβ level [19]. This discrepancy prompted us to revisit this issue. In the present study, we showed that the level of C99 in APP A673T cells was comparable to that detected in APP WT cells, despite a significant reduction in sAPPβ. Currently, we do not have clear interpretations of the discrepancy between the amount of sAPPβ and C99 levels in APP A673T cells. However, it is interesting to note that the generation rate of C83 was distinct from that of C99, although the generation curves of sAPPα and sAPPβ had some resemblance to each other (see Fig. 2b and c). This suggests that the levels of those soluble APP fragments do not reflect the levels of their stubs in cells precisely.

Our reconstituted β-secretase assay demonstrated that the APP633–685 fragment carrying A673T was cleaved by β-secretase, as was the WT APP638–685 fragment. This result was consistent with our quantitative analyses of C99 levels on A673T cells; however, it was inconsistent with the result of the cell-based assay of sAPPβ as reported previously [9, 18]. One possible interpretation for the inconsistent results between the reconstituted β-secretase assay and the cell-based assay is that APP A673T is preferentially processed by an unknown enzyme, leading to a reduced level of sAPPβ from APP A673T cells. Recently, Willem and colleagues reported η-secretase as a novel APP-processing enzyme that produces a high-molecular-weight carboxyl terminal fragment of APP (CTFη), which can be processed into C83 and C99 by α- and β-secretases, respectively [34]. One can safely say that η-secretase cleaves APP A673T preferentially. If so, APP A673T cells produce a lesser amount of sAPPβ, but an equal level of C99, compared with APP WT cells. Alternatively, η-secretase may preferentially cleave sAPPβ produced from APP A673T cells.

We have shown that C99 A2T cells also release a lower amount of Aβ compared with C99 WT cells. This is direct evidence that the A2T substitution on C99 alters Aβ production. This was also observed for the other cell lines. Our western blotting assay using 6E10 and E50 revealed that Aβ A2T and C99 A2T were transferred onto nitrocellulose, as were Aβ WT and C99 WT. Our data demonstrated that 82E1 failed to recognize Aβ A2T and C99 A2T on western blot and to capture the C99 A2T substrate on immunoprecipitation. This indicates that our evaluation by western blotting is reliable and that C99 A2T is an inefficient substrate for Aβ production in cells. Importantly, the amount of the p3 peptide in the media of C99 WT and C99 A2T cells was indistinctive, which suggests that the A2T substitution affects the γ-cleavage of C99, but not that of C83. We also found that the A2T substitution in C99 caused no accumulation of intracellular Aβ; rather, it reduced the level of intracellular Aβ. Our data demonstrated that reduced Aβ levels in the medium of C99 A2T cells were caused by impaired γ-cleavage of C99 A2T in cells.

We expected that C99 A2T would be an inefficient substrate for γ-secretase even in the CHAPSO-solubilized γ-secretase assay. However, we observed no significant reduction in Aβ production from the recombinant C99 A2T substrate, and an interaction with γ-secretase components in the CHAPSO-solubilized state. This implies that membrane solubilization disrupts the intact distribution of C99 A2T, which allows free collision between the enzyme and the substrate in the solution. To mimic the cell-based assay in a biochemical analysis, we chose a cell-free assay that used the microsomal fraction of cells. This approach provides an assessment of Aβ production in intact membranes. As expected, the cell-free assay reproduced the altered Aβ production in a membrane fraction of C99 A2T cells. This suggests that the subcellular distribution of C99 A2T is altered, and that this redistribution reduces the interaction between C99 A2T and γ-secretase.

## Conclusion

This report indicates that the C99 level in APP A673T cells is comparable to that in APP WT cells despite a significant reduction in released sAPPβ level, suggesting vulnerable correlation between levels of sAPPβ and C99 in cells. Our data demonstrate that the A673T mutation in C99 impairs γ-cleavage the in cell-based assay. Assessment of observed results in vivo may be crucial to elucidate the protective mechanism of A673T mutation against AD.

## Availability of supporting data

The data set supporting the results of this article are included within the article and its additional files.

## Additional file

Additional file 1: Figure S1. Immunoreactivity of anti-Aβ antibodies. Recognition sites of anti-Aβ antibodies in the human C99/Aβ sequence (A). A recombinant C99-FLAG containing the A2T substitution was subjected to Western blotting using anti-Aβ antibodies (B). Aβ40 containing the A2T substitution was subjected to Western blotting using anti-Aβ antibodies (C). 82E1 failed to recognize C99-FLAG and Aβ40 containing the A2T substitution. **Figure S2.** Immunoprecipitation of C99 A2T in APP A673T cells. CHO cells expressing APP A673T were solubilized in 1% NP40 and subjected to immunoprecipitation with 6E10 or 82E1 and to Western blotting, to visualize C99 (upper panel) (A). Immunoprecipitation with 6E10 proved that the level of C99 (C99 A2T) in APP A673T cells was indistinctive of that observed in APP WT cells; however, 82E1 failed to capture C99 A2T (upper and lower panels). These data indicate that the amount of C99 in APP A673T cells was comparable to that detected in APP WT cells. Arrowheads, IgG; * degradation products of APP.CHO cells expressing APP A673T were solubilized in 1% NP40 and subjected to immunoprecipitation with anti-FLAG M2 antibody and to Western blotting (B). Anti-FLAG M2 antibody visualized equal levels of C99 in APP WT and A673T cells (left panel). The blot was reprobed with 82E1 after stripping anti-FLAG M2 (right panel). 82E1 failed to visualize C99 from APP A673T cells, although remnant bands were detected on the blot. These data indicate that the amount of C99 in APP A673T cells was comparable to that detected in APP WT cells. Arrowheads, IgG. **Figure S3.** Cells expressing APP A673T and C99 A2T exhibited decreased extracellular Aβ production. HEK293, Neuro 2a, and CHO cells were transfected with the APP A673T or C99 A2T construct. Conditioned media were subjected to Western blotting, to visualize and quantify extracellular Aβ, as in Fig. 2A. HEK, HEK293; N2a, Neuro 2a. Data represent means ± SD of three independent experiments. * P < 0.05; ** P < 0.005 (unpaired t-test). **Figure S4.** No effect of the C99 A2T substitution on the subcellular distribution of γ-secretase components. γ-Secretase components were enriched in lipid raft fractions, as well as caveolin and flotilin raft markers (#4 - #6) (A). The C99 substrate also localized in the fractions, in part; however, C99 A2T was distributed in denser fractions (#5 and #6) of raft fractions compared with C99 WT (#4, #5, and #6) (see Fig. 1B and C). mNCT, mature nicastrin. Quantitative analysis of the distribution of g-secretase components and C99 substrates (B). C99 A2T was distributed in denser fractions (#5 and #6) compared with C99 WT (#4, #5, and #6) (see Fig. 1B and C). This indicated that the level of C99 A2T that was colocalized with g-secretase was decreased. Data represent means ± SD of three independent experiments. No significant difference was observed between C99 WT and C99 A2T (unpaired t-test). Individual C99 blots of each experiment (C). Levels of C99 A2T in fraction 4 were lower than those in fraction 6. However levels of C99 WT in fraction 4 were equivalent or higher than those in fraction 6. **Figure S5.** C99 in membrane, cytosolic and nuclear fractions. C99 cells are homogenized PEPES buffer containing 250 mM sucrose. Postnuclear fraction was subjected to ultracentrifugation at 100,000 g for 1 h. The pellet was resuspended in the original volume of the PIPES buffer and referred to as membrane fraction. Each fraction was subjected to western blotting to visualize C99 (A). Band intensities of C99, band A and band B in membrane (Mem), cytosolic (Cyto) and nuclear (Nuc) fractions were quantified (B). No prominent difference was observed between C99 WT and C99 A2T. (PDF 935 kb)

## Abbreviations

AD: Alzheimer’s disease
AICD: APP intracellular domain
APP: amyloid precursor protein
Aβ: β-amyloid protein
C99: β-carboxyl fragment of APP
FAD: familial AD
IRES: internal ribosome entry site
sAPPβ: soluble APP fragment processed by β-secretase
WT: wild-type
