# Supplementary material for: The A673T mutation in the amyloid precursor protein reduces the production of β-amyloid protein from its β-carboxyl terminal fragment in cells
Source: Acta Neuropathol Commun. 2015 Nov 4;3:66. doi: 10.1186/s40478-015-0247-6 (PMC4632685; doi:10.1186/s40478-015-0247-6)
Supplement: Additional file 1: Figure S1. — Immunoreactivity of anti-Aβ antibodies. Recognition sites of anti-Aβ antibodies in the human C99/Aβ sequence (A). A recombinant C99-FLAG containing the A2T substitution was subjected to Western blotting using anti-Aβ antibodies (B). Aβ40 containing the A2T substitution was subjected to Western blotting using anti-Aβ antibodies (C). 82E1 failed to recognize C99-FLAG and Aβ40 containing the A2T substitution. Figure S2. Immunoprecipitation of C99 A2T in APP A673T cells. CHO cells expressing APP A673T were solubilized in 1% NP40 and subjected to immunoprecipitation with 6E10 or 82E1 and to Western blotting, to visualize C99 (upper panel) (A). Immunoprecipitation with 6E10 proved that the level of C99 (C99 A2T) in APP A673T cells was indistinctive of that observed in APP WT cells; however, 82E1 failed to capture C99 A2T (upper and lower panels). These data indicate that the amount of C99 in APP A673T cells was comparable to that detected in APP WT cells. Arrowheads, IgG; * degradation products of APP.CHO cells expressing APP A673T were solubilized in 1% NP40 and subjected to immunoprecipitation with anti-FLAG M2 antibody and to Western blotting (B). Anti-FLAG M2 antibody visualized equal levels of C99 in APP WT and A673T cells (left panel). The blot was reprobed with 82E1 after stripping anti-FLAG M2 (right panel). 82E1 failed to visualize C99 from APP A673T cells, although remnant bands were detected on the blot. These data indicate that the amount of C99 in APP A673T cells was comparable to that detected in APP WT cells. Arrowheads, IgG. Figure S3. Cells expressing APP A673T and C99 A2T exhibited decreased extracellular Aβ production. HEK293, Neuro 2a, and CHO cells were transfected with the APP A673T or C99 A2T construct. Conditioned media were subjected to Western blotting, to visualize and quantify extracellular Aβ, as in Fig. 2A. HEK, HEK293; N2a, Neuro 2a. Data represent means ± SD of three independent experiments. * P < 0.05; ** P < 0.005 (unpaired t-test). Figur [file 40478_2015_247_MOESM1_ESM.pdf]

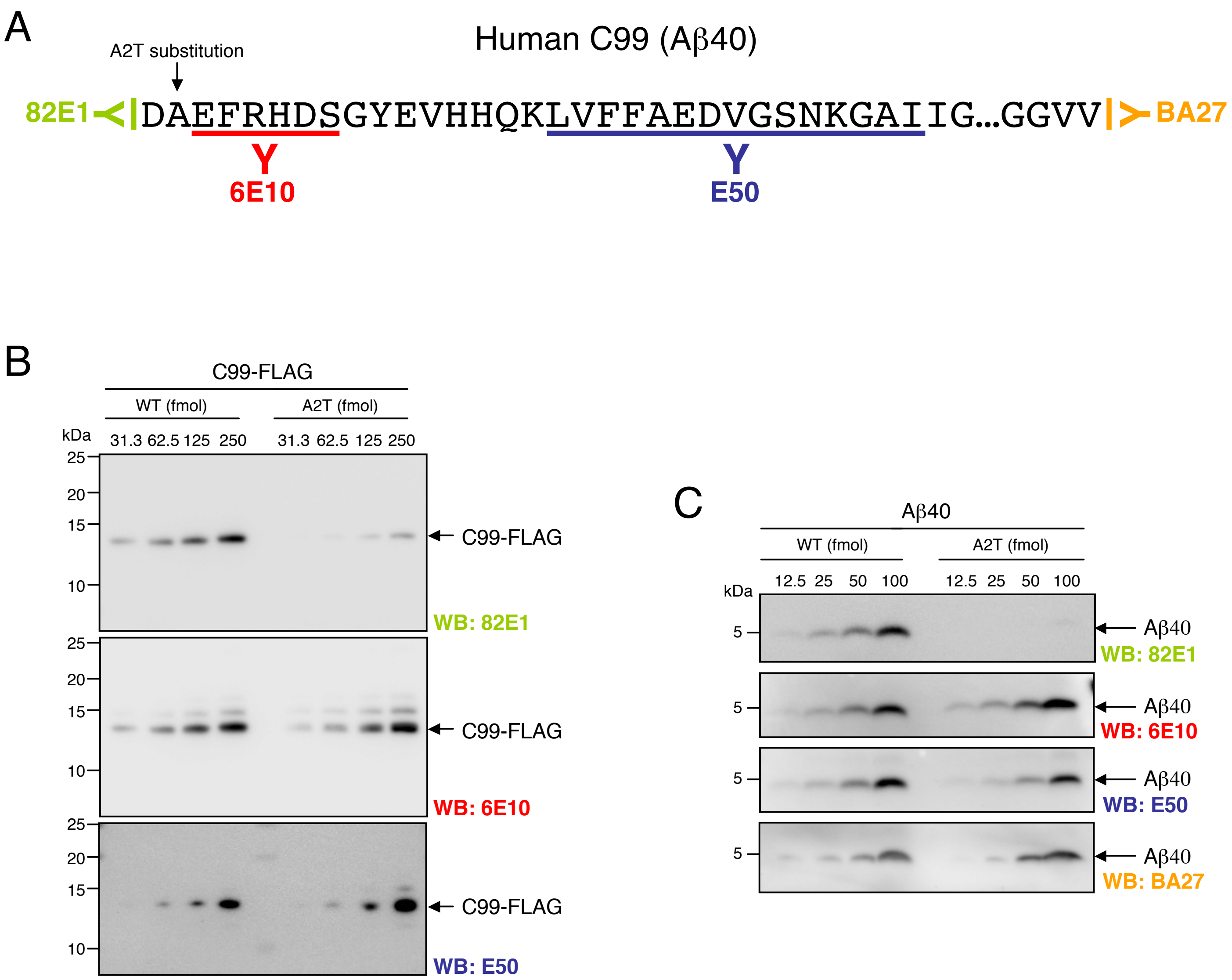

Supplementary Figure 1. Immunoreactivity of anti-Aβ antibodies. Recognition sites of anti-Aβ antibodies in the human C99/Aβ sequence (A). A recombinant C99-FLAG containing the A2T substitution was subjected to Western blotting using anti-Aβ antibodies (B). Aβ40 containing the A2T substitution was subjected to Western blotting using anti-Aβ antibodies (C). 82E1 failed to recognize C99-FLAG and Aβ40 containing the A2T substitution.

A

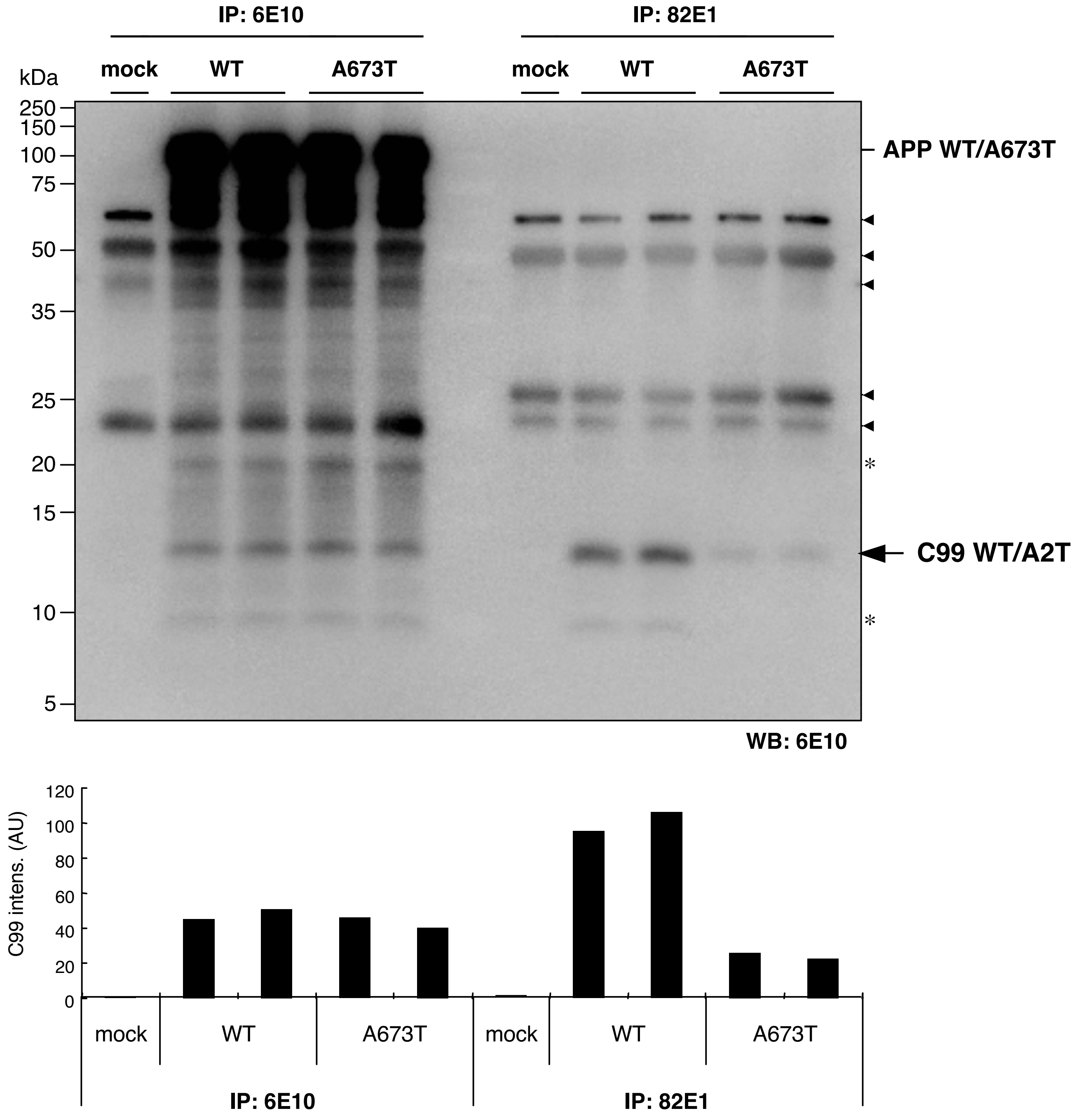

Supplementary Figure 2. Immunoprecipitation of C99 A2T in APP A673T cells. A. CHO cells expressing APP A673T were solubilized in 1% NP40 and subjected to immunoprecipitation with 6E10 or 82E1 and to Western blotting, to visualize C99 (upper panel). Immunoprecipitation with 6E10 proved that the level of C99 (C99 A2T) in APP A673T cells was indistinctive of that observed in APP WT cells; however, 82E1 failed to capture C99 A2T (upper and lower panels). These data indicate that the amount of C99 in APP A673T cells was comparable to that detected in APP WT cells. Arrowheads, IgG; * degradation products of APP.

B

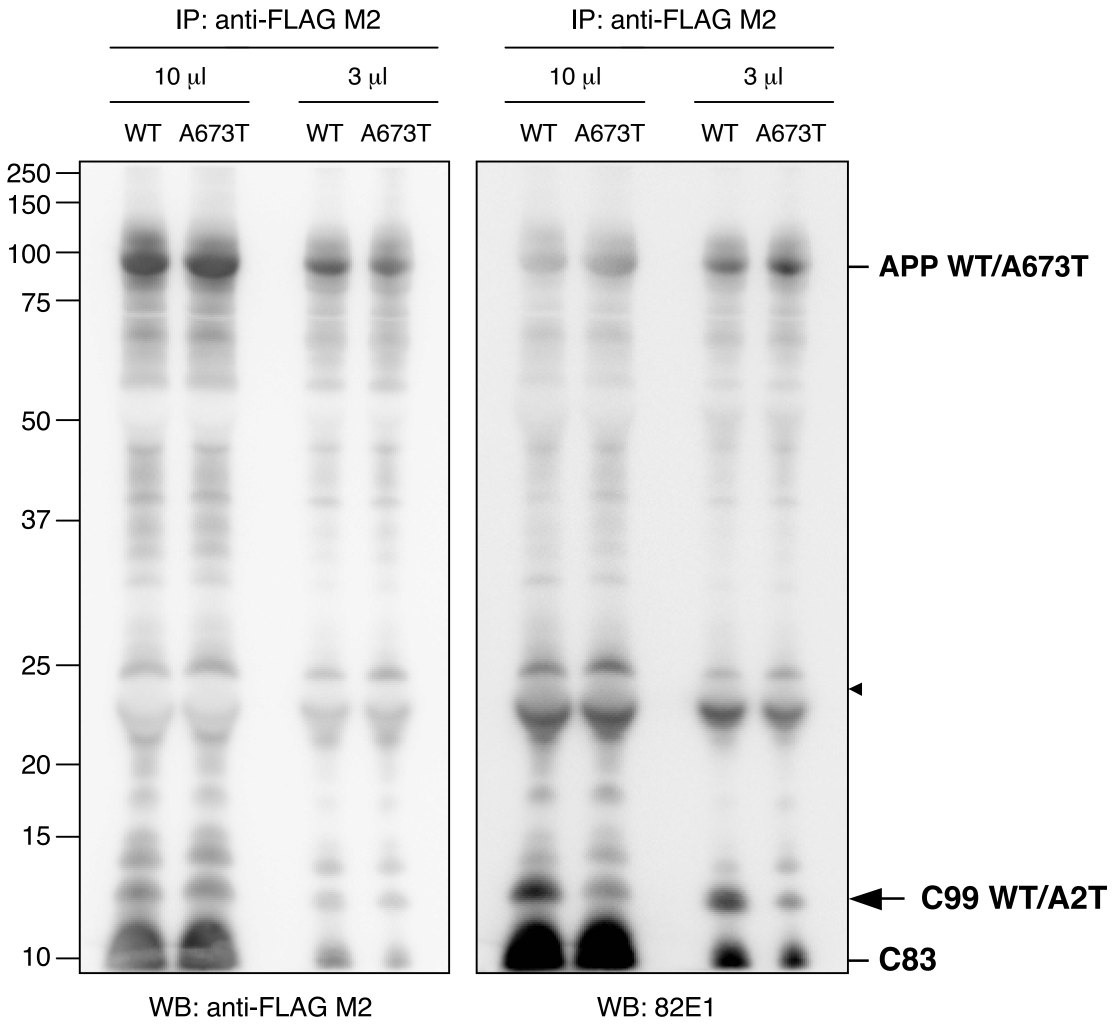

Supplementary Figure 2. Immunoprecipitation of C99 A2T in APP A673T cells (continued). B. CHO cells expressing APP A673T were solubilized in 1% NP40 and subjected to immunoprecipitation with anti-FLAG M2 antibody and to Western blotting. Anti-FLAG M2 antibody visualized equal levels of C99 in APP WT and A673T cells (left panel). The blot was reprobed with 82E1 after stripping anti-FLAG M2 (right panel). 82E1 failed to visualize C99 from APP A673T cells, although remnant bands were detected on the blot. These data indicate that the amount of C99 in APP A673T cells was comparable to that detected in APP WT cells. Arrowheads, IgG.

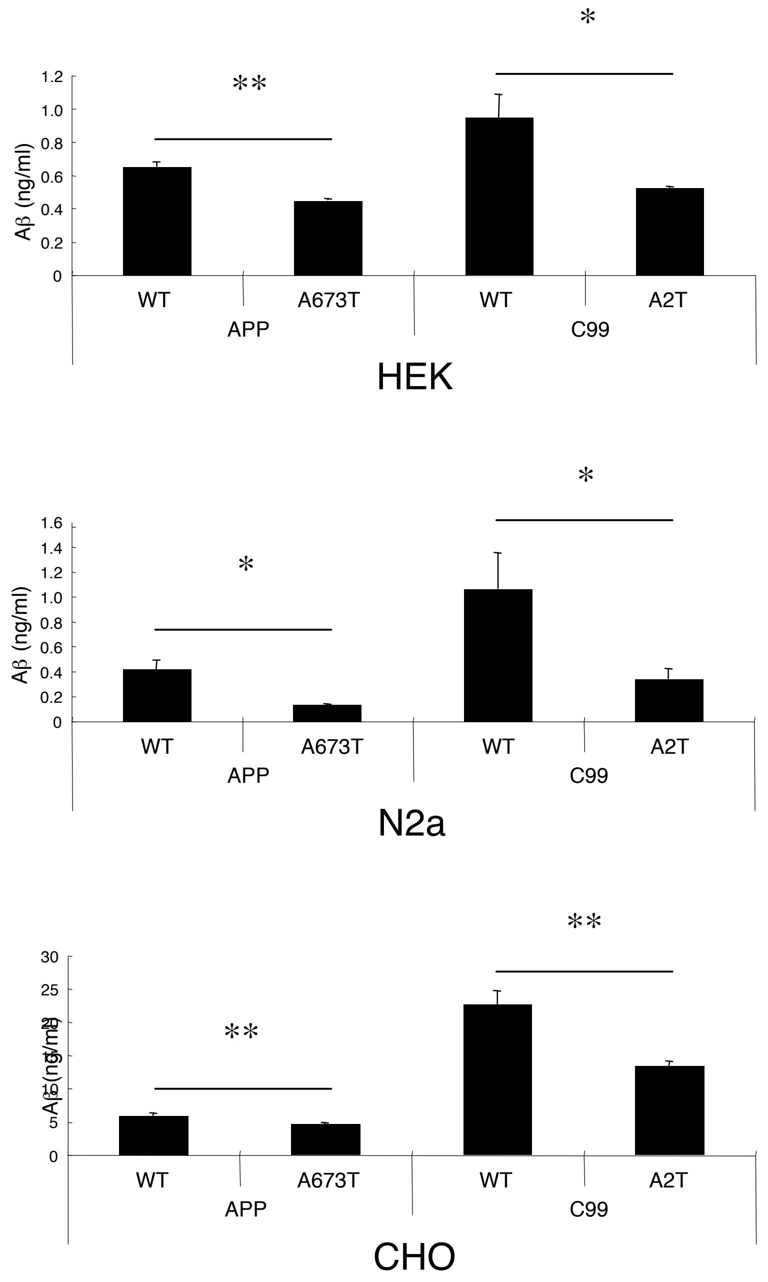

Supplementary Figure 3. Cells expressing APP A673T and C99 A2T exhibited decreased extracellular Aβ production. HEK293, Neuro 2a, and CHO cells were transfected with the APP A673T or C99 A2T construct. Conditioned media were subjected to Western blotting, to visualize and quantify extracellular Aβ, as in Fig. 2A. HEK, HEK293; N2a, Neuro 2a. Data represent means ± SD of three independent experiments. * $P < 0.05$; ** $P < 0.005$ (unpaired *t*-test).

A

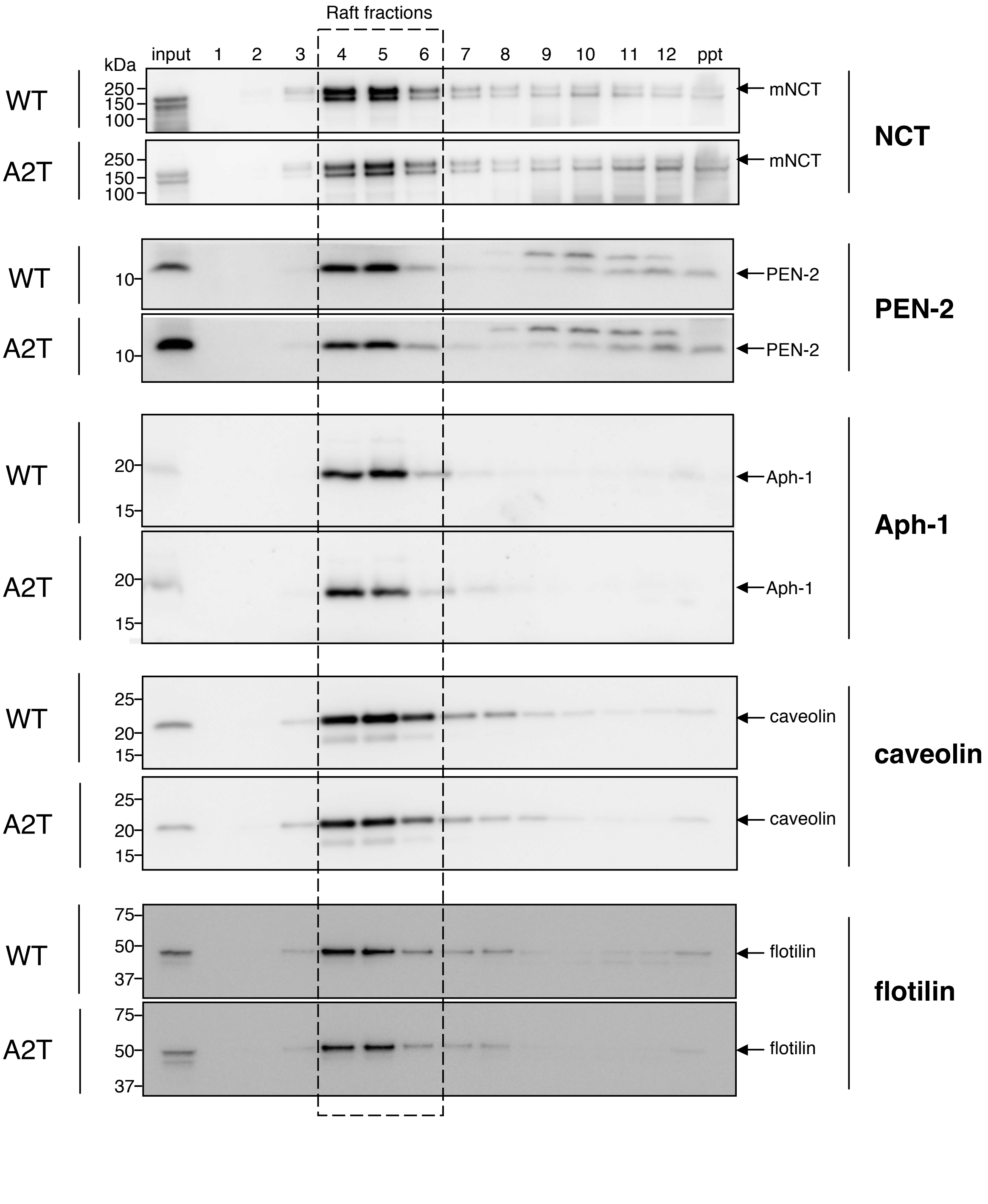

Supplementary Figure 4. No effect of the C99 A2T substitution on the subcellular distribution of γ-secretase components. γ-Secretase components were enriched in lipid raft fractions, as well as caveolin and flotilin raft markers (#4 - #6) (A). The C99 substrate also localized in the fractions, in part; however, C99 A2T was distributed in denser fractions (#5 and #6) of raft fractions compared with C99 WT (#4, #5, and #6) (see Fig. 1B and C). mNCT, mature nicastrin.

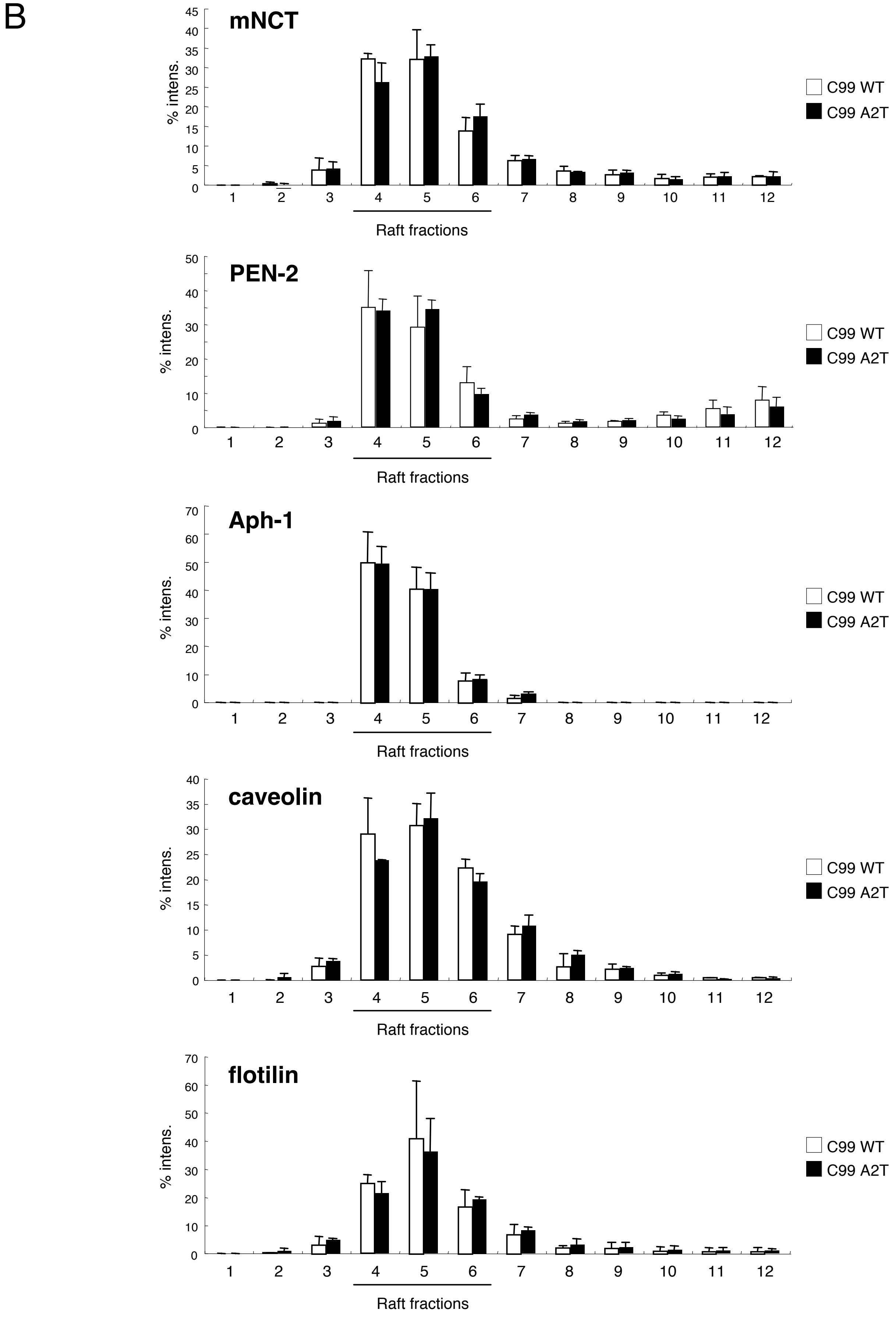

Supplementary Figure 4. No effect of the C99 A2T substitution on the subcellular distribution of γ-secretase components (continued). Quantitative analysis of the distribution of γ-secretase components and C99 substrates (B). C99 A2T was distributed in denser fractions (#5 and #6) compared with C99 WT (#4, #5, and #6) (see Fig. 1B and C). This indicated that the level of C99 A2T that was colocalized with γ-secretase was decreased. Data represent means ± SD of three independent experiments. No significant difference was observed between C99 WT and C99 A2T (unpaired *t*-test).

C

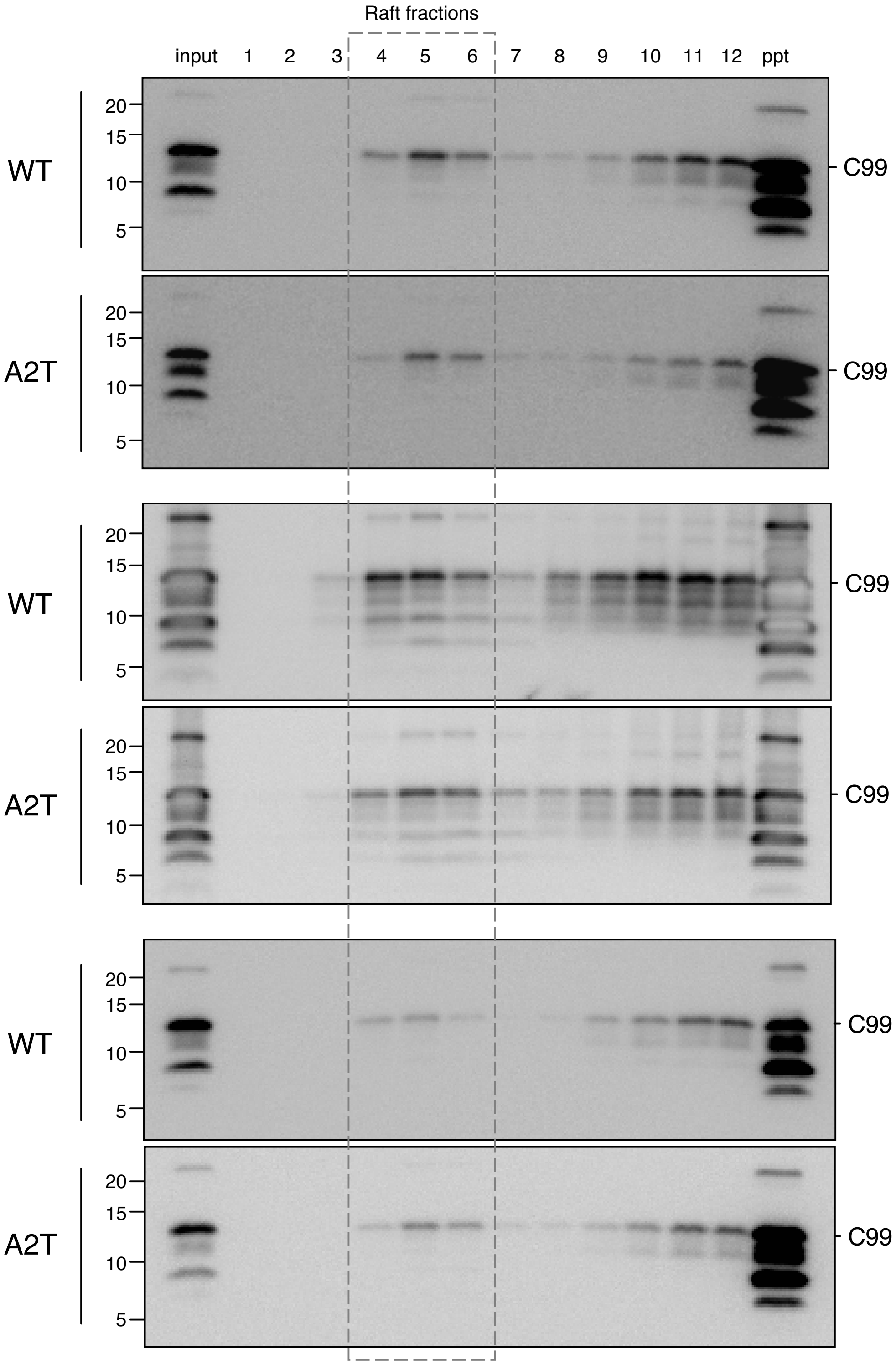

Supplementary Figure 4. No effect of the C99 A2T substitution on the subcellular distribution of γ-secretase components (continued). Individual C99 blots of each experiment (C). Levels of C99 A2T in fraction 4 were lower than those in fraction 6. However levels of C99 WT in fraction 4 were equivalent or higher than those in fraction 6.

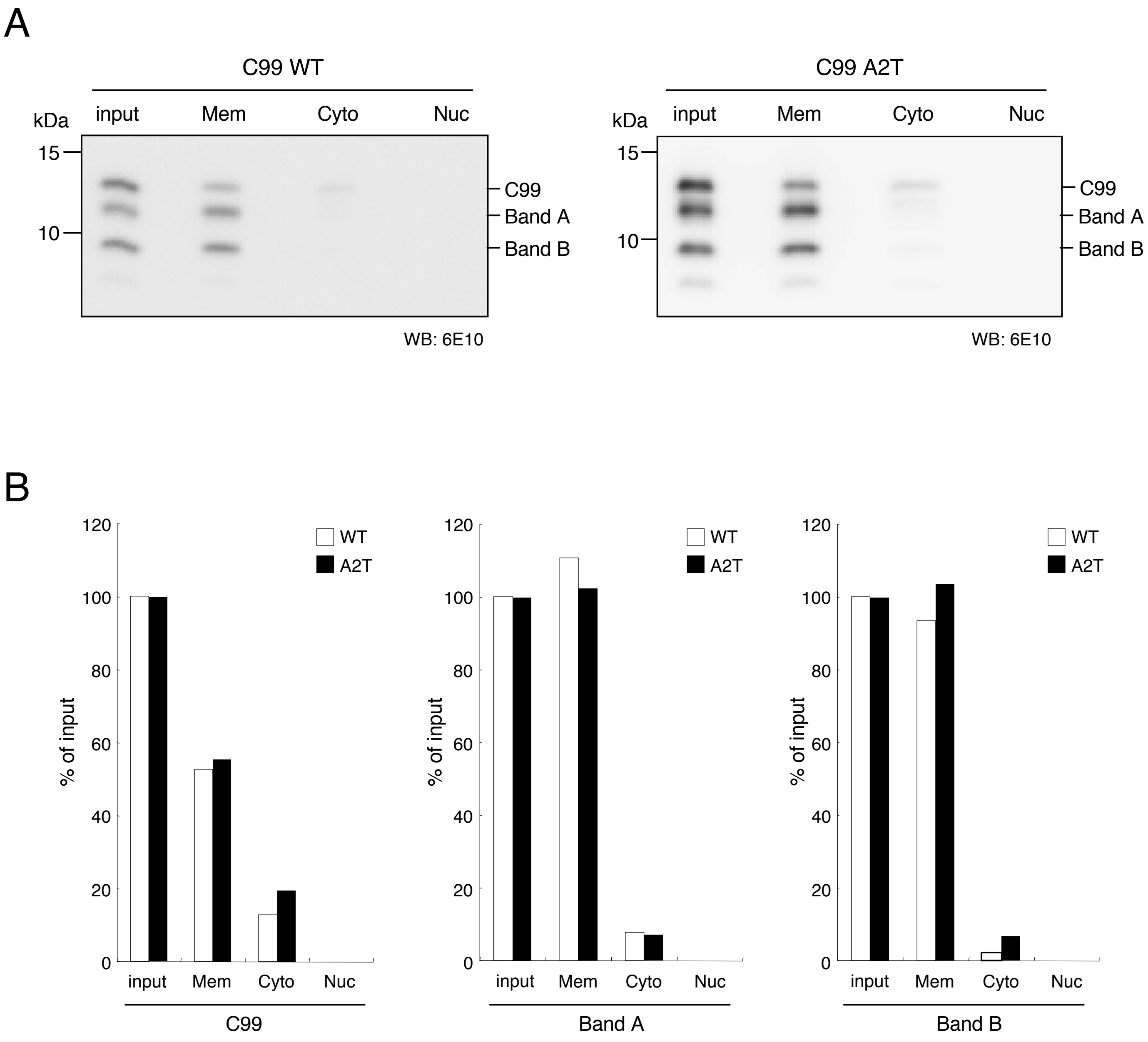

Supplementary Figure 5. C99 in membrane, cytosolic and nuclear fractions. C99 cells are homogenized PEPES buffer containing 250 mM sucrose. Postnuclear fraction was subjected to ultracentrifugation at 100,000 *g* for 1 h. The pellet was resuspended in the original volume of the PIPES buffer and referred to as membrane fraction. Each fraction was subjected to western blotting to visualize C99 (A). Band intensities of C99, band A and band B in membrane (Mem), cytosolic (Cyto) and nuclear (Nuc) fractions were quantified (B). No prominent difference was observed between C99 WT and C99 A2T.
